# Neonatal immune response to rhinovirus A16 has diminished dendritic cell function and increased B cell activation

**DOI:** 10.1371/journal.pone.0180664

**Published:** 2017-10-18

**Authors:** Amanda Barlow-Anacker, Yury Bochkov, James Gern, Christine M. Seroogy

**Affiliations:** Department of Pediatrics, Division of Allergy, Immunology, & Rheumatology, University of Wisconsin School of Medicine and Public Health, Madison, WI, United States of America; Imperial College London, UNITED KINGDOM

## Abstract

**Background:**

Rhinovirus infections during infancy account for the majority of respiratory illness health care utilization and are an associated risk factor for subsequent development of allergic asthma. Neonatal type I interferon production is diminished compared to adults after stimulation with TLR agonists. However, broad profiling of immune cell responses to infectious rhinovirus has not been undertaken and we hypothesized that additional immune differences can be identified in neonates. In this study, we undertook a comparative analysis of neonatal and adult blood immune cell responses after *in vitro* incubation with infectious RV-A16 for 6 and 24 hours.

**Methods:**

Intracellular proinflammatory and type I interferon cytokines along with expression of surface co-stimulatory and maturation markers were measured using multi-parameter flow cytometry.

**Results:**

Both circulating myeloid dendritic cell (mDC) and plasmacytoid dendritic cell (pDC) frequency were lower in cord blood. Qualitative and quantitative plasmacytoid dendritic cell IFN-alpha + TNF- alpha responses to rhinovirus were significantly lower in cord pDCs. In cord blood samples, the majority of responsive pDCs were single-positive TNF-alpha producing cells, whereas in adult samples rhinovirus increased double-positive TNF-alpha+IFN-alpha+ pDCs. Rhinovirus upregulated activation and maturation markers on monocytes, mDCs, pDCs, and B cells, but CD40+CD86+ monocytes, mDCs, and pDCs cells were significantly higher in adult samples compared to cord samples. Surprisingly, rhinovirus increased CD40+CD86+ B cells to a significantly greater extent in cord samples compared to adults.

**Conclusions:**

These findings define a number of cell-specific differences in neonatal responses to rhinovirus. This differential age-related immune response to RV may have implications for the immune correlates of protection to viral respiratory illness burden and determination of potential biomarkers for asthma risk.

## Introduction

Neonates are more susceptible to respiratory viral illnesses partly due to immaturity of both the adaptive and innate immune systems. Viral respiratory illnesses account for considerable morbidity in early life, and in a subset of babies likely contributes to the subsequent development of chronic airway disease [1, 2]. The immune mechanisms that protect against viral respiratory illnesses in early life remain poorly defined. Previous studies primarily focused on responses of plasmacytoid dendritic cells (pDC) type I IFN [3]. These studies have largely relied on use of isolated toll-like receptor (TLR) agonists, as opposed to the intact and more complex viral pathogens for characterization of neonatal anti-viral innate immune responses.

Rhinoviruses (RV) are the most commonly detected respiratory viral pathogens in infants with medically-attended respiratory illnesses [4, 5]. Early in life symptomatic viral respiratory illnesses, especially RV illnesses, are associated with subsequent development of asthma in children [6–10]. RV can directly or indirectly activate immune cells [11]. Previous studies, using adult blood cells, have demonstrated that blood cell transcriptional responses to RV correlate with disease severity and recapitulate in vivo innate immune response pathways, thus supporting the use of circulating blood cells to study RV immune responses ex vivo[12, 13].

Given the greater susceptibility of infants to RV illnesses, we hypothesized that, in addition to diminished type I IFN response, neonates will have other anti-RV maturational immune differences compared to adults. To test this hypothesis, we stimulated cord and adult blood mononuclear cells with a major group RV, and used multi-parameter flow cytometry to define the phenotypic and functional differences in monocytes, mDCs, pDCs and B cells.

## Materials and methods

### Human subjects

Deidentified cord blood was obtained from full term infants delivered via scheduled c-section in sodium heparin tubes. Deidentified adult blood was obtained from healthy adults in sodium heparin tubes. Samples were collected between September 2014-August 2016. All studies were approved by the Institutional Review Board at the University of Wisconsin and Meriter Hospital, Madison WI.

### Rhinovirus A16 (RV-A16) preparation and purification

HeLa-adapted RV-A16 strain was grown in H1-HeLa cell suspension culture (ATCC CRL-1958) [14]. Virions were purified in two steps by centrifugation through a sucrose cushion and a sucrose gradient as previously described [15, 16]. The titer of the purified virus stock was determined by plaque assay in HeLa cell monolayers and calculated in plaque-forming units (PFU).

### Isolation of blood mononuclear cells and stimulation

Peripheral blood mononuclear cells were isolated using Lymphocyte Separation Medium (Mediatech Inc) according to manufacturer’s instructions within 24 hours of sample collection.

We adapted a well-standardized assay platform for measurement of RV immune responses [17], and adhered to published principles to control for potential performance variables and optimize test precision [18–22]. Pilot dose-response experiments were run to determine the RV-A16 (referred to as RV in remaining text) multiplicity of infection (MOI) that provided a reproducibly detectable type I IFN response in adult samples. Cells were incubated in standardized stimulation plates with medium alone or RV at a MOI of 20 PFU per cell for both 6 hours and 24 hours at 37°C 5% CO_2_. For the 6 hours assay, brefeldin A was added after the initial 3 hours incubation. After indicated incubation time, cells were pelleted, resuspended in FACS Lysing solution (BD Biosciences) and stored at -80°C until processed for flow cytometry staining and acquisition.

### Flow cytometry

Cell staining used two multi-parameter flow cytometry assay panels optimized to interrogate innate cell maturation and function S1 and S2 Tables. Acquisition was performed on a Fortessa (for the 24 hours flow panel exclusively) or LSR II (for the 6 hours flow panel exclusively) (BD Biosciences) cytometers with daily machine standardization with CS&T beads (BD Biosciences) and calibration using manufacturer’s Cytometer Settings and Tracking calibration software. Each flow run was normalized by the use of Sphero™ Rainbow calibration particles (Spherotech) to ensure that identical voltages were used for acquisitions of all fluorescent channels on all samples. Data were analyzed using FlowJo v.10.1 (FlowJo LLC) in a semi-automated manner with a combination of manual gating (using the unstimulated sample and fluorescence minus one (FMOs) to determine positive populations), templates, and boolean gating for determination of polyfunctionality (production of more than one cytokine/cell). Variables include frequency of target population, geometric mean fluorescence intensity (gMFI), and integrated gMFI (igMFI) calculated as previously described [23]. Data may be accessed from http://www.immport.org/immport-open/public/home/studySearch with study accession SDY1115.

### Statistical analysis

Power calculations using published pDC function results with RSV stimulation determined our study sample size was adequate for detection of differences with a power of 80% at p = 0.05 level of significance[1]. Statistical comparison between different subject groups was performed using the nonparametric Mann–Whitney test, Prism 7 (GraphPad Software, Inc). A p value ≤ 0.05 was considered statistically significant.

## Results

### Circulating dendritic cell frequency varies between cord and adult blood

**Fig 1:**
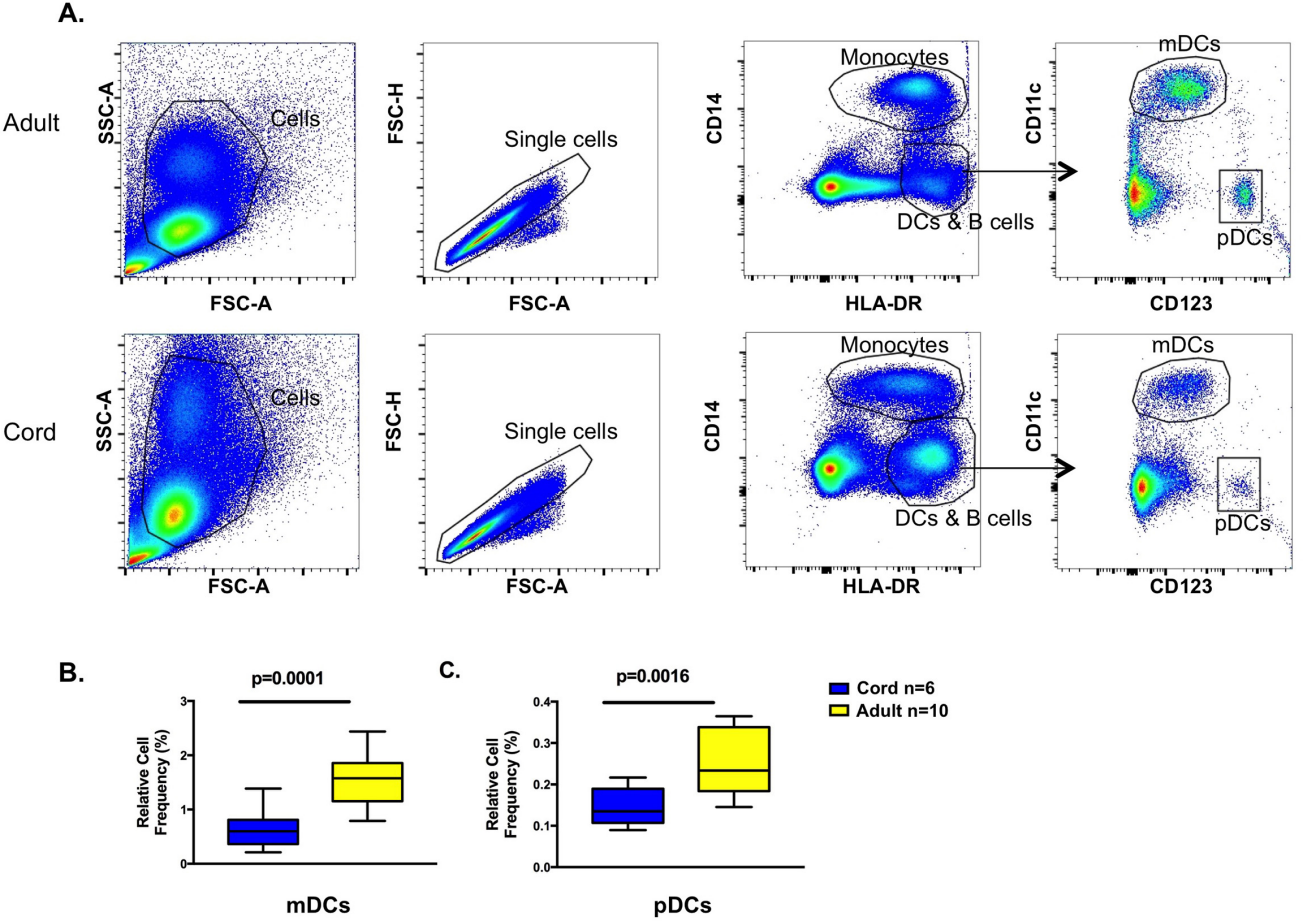
Gating strategy and dendritic cell frequency. **A.** Gating strategy for determination of monocytes and dendritic cells. Representative adult (top) and cord (bottom) subjects shown. **B.** Box-and-whisker plot of mDC frequency. **C.** Box-and-whisker plot pDC frequency. mDC and pDC frequency is percentage of gated single cells from unstimulated samples.

Cell frequency was determined after density gradient centrifugation for isolation of mononuclear cells. The gating strategy for identification of cell lineages is shown (Fig 1A). Both the myeloid (mDC) and plasmacytoid (pDC) dendritic cell frequencies were higher in adult compared to cord samples (Fig 1B & 1C). There were no significant differences in monocyte or B cell frequencies between cord and adult subjects (data not shown).

### Cord pDC cell cytokine responses are diminished compared to adult pDC with RV exposure

**Fig 2:**
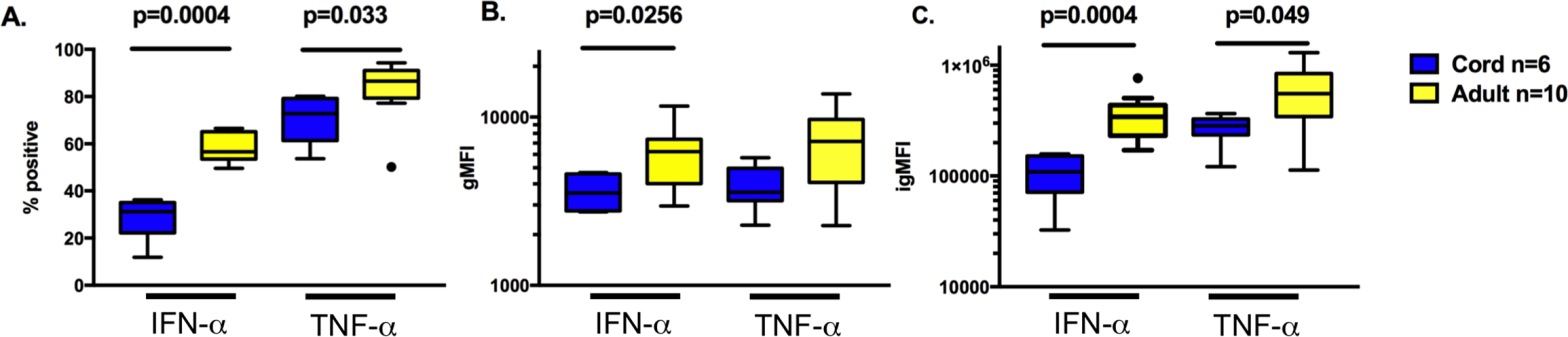
Plasmacytoid dendritic cell cytokine production after RV stimulation. After 6 hours exposure to RV and from pDC gate, box-and-whisker plot: **A.** Percent positive IFN-α and TNF-α producing cells. **B.** gMFI of cytokine **C.** igMFI (see materials and methods) of cytokine producing cells.

**Fig 3:**
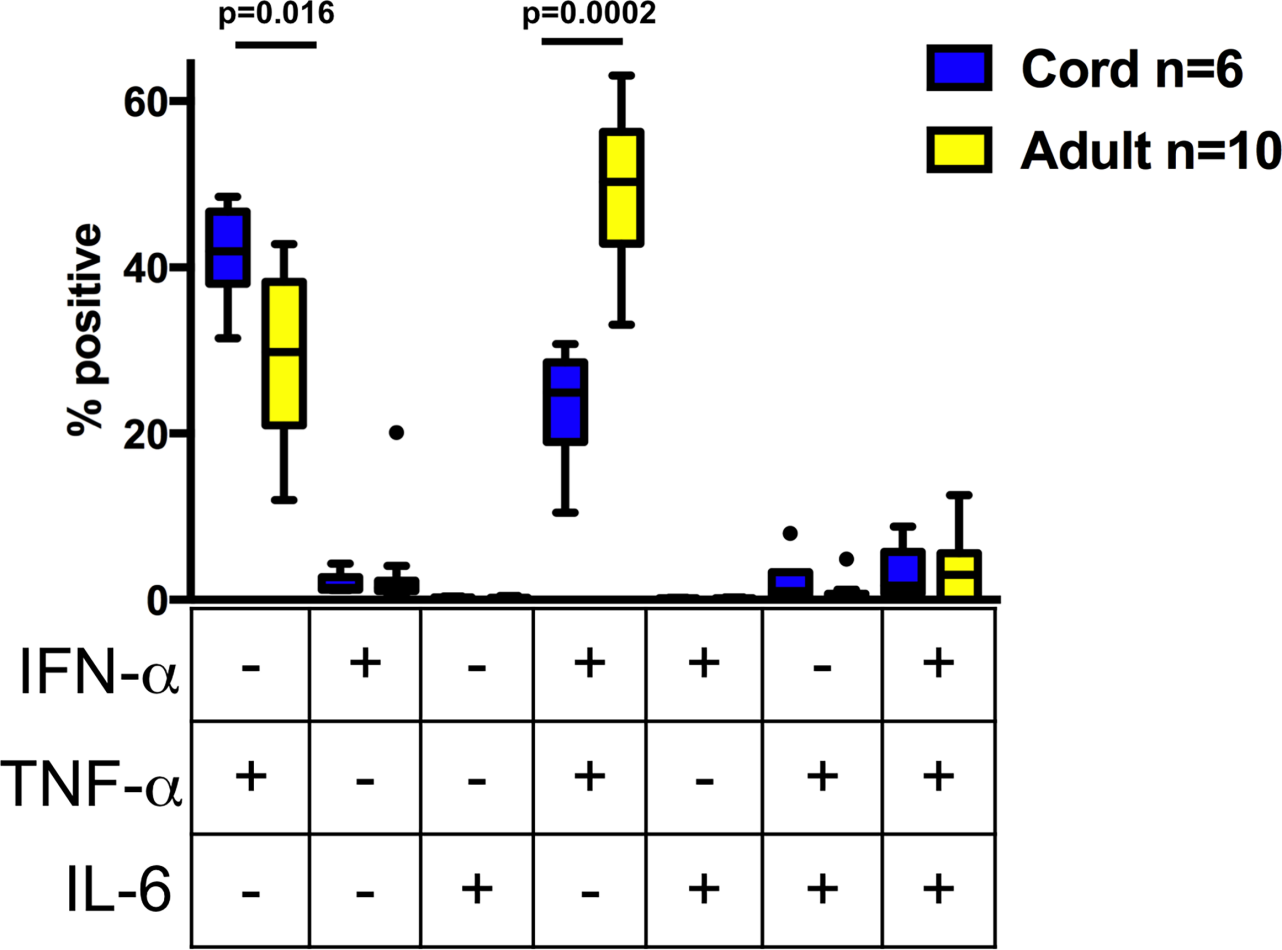
Plasmacytoid dendritic cell polyfunctionality after RV stimulation. Box-and-whisker plot from pDC gate, frequency of one, two, and three cytokine producing cells using boolean gating after 6 hours exposure to RV. Significant p values are shown in the graph, there was no significant difference between cord and adult samples for triple positive cytokine producing cells.

To determine cytokine production on a per-cell-basis, intracellular cytokine staining was performed after 6 hours of stimulation with RV as described in materials and methods section. Following RV stimulation, pDCs were the only cell lineage with detectable cytokine production above background in all samples. Significantly higher qualitative (% positive), quantitative (gMFI), and the combination (igMFI), differences were found in adult pDCs for both IFN-alpha and TNF-alpha cytokines compared to cord pDCs (Fig 2A–2C). Adult pDCs had significantly higher IFN-alpha+TNF-alpha + frequency compared to cord pDCs, whereas the majority of cytokine producing cord pDCs were TNF-alpha+ only (Fig 3). In our assay system, IL-6 producing cells were detectable at a low level and did not differ between adult and cord samples (Fig 3).

### Cord monocytes and DCs have diminished activation and maturation compared to adult cells with RV exposure

**Fig 4:**
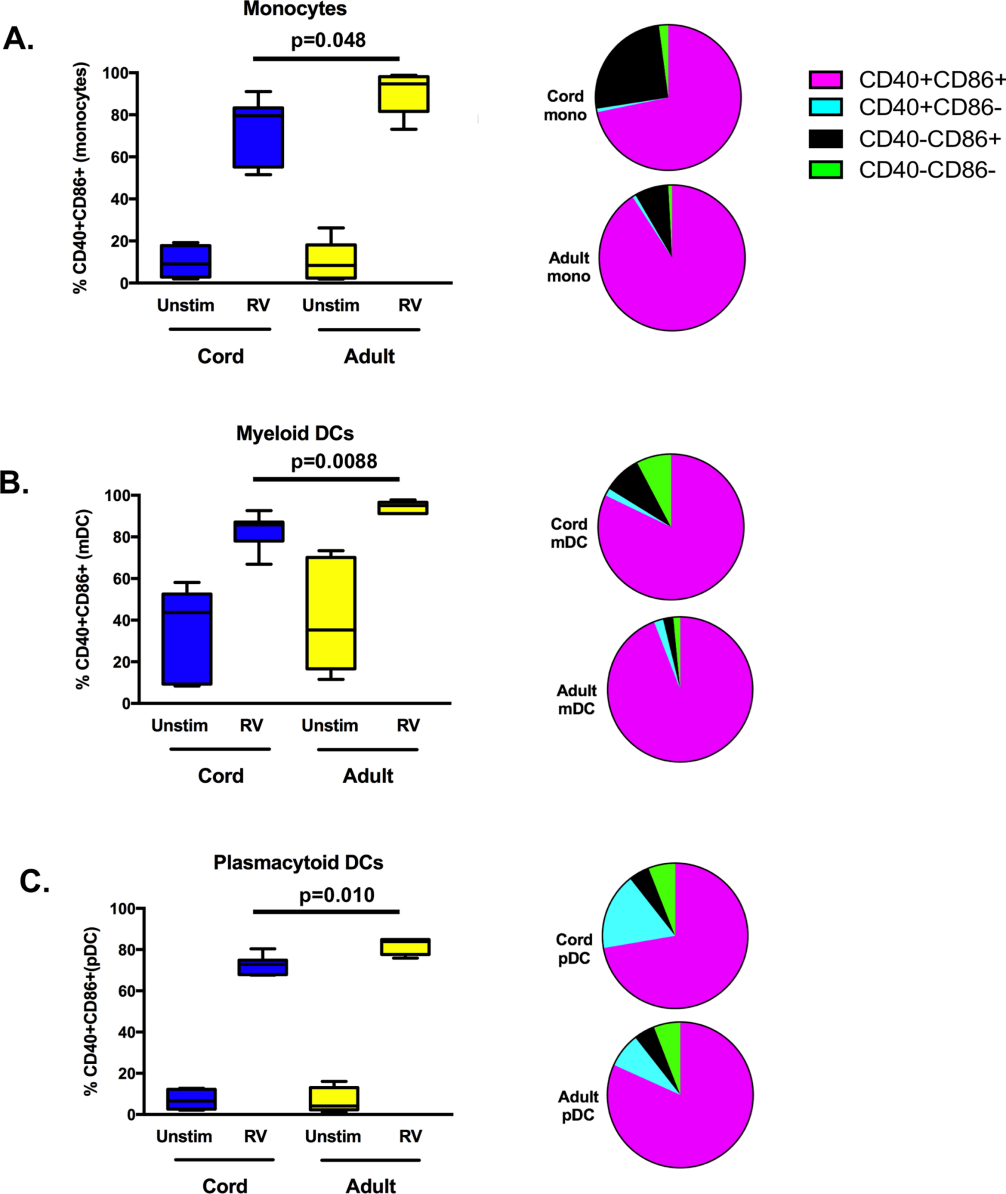
Costimulatory marker expression after RV stimulation. Frequency of double positive (CD40+CD86+) cells box-and-whisker plot on left and parts of whole graph of varied phenotypes on right after 24 hours exposure to RV or unstimulated (Unstim). **A.** Monocytes. **B.** mDC. **C.** pDC. Cord n = 7; Adult n = 5. Significant p values are shown in the graph, unstimulated comparisons between adult and cord samples were not significant.

No significant differences were noted in unstimulated CD40+CD86+ monocytes, mDCs, or pDCs between cord and adult samples (Fig 4A–4C). After 24 hours stimulation with RV, the majority of monocytes, mDCs, and pDCs express CD40, CD86, or both in both adult and cord samples (Fig 4A–4C). Adult cells demonstrated a significantly higher frequency of CD40+CD86+ monocytes, pDCs and mDCs compared to cord cells (Fig 4A–4C and Table 1). Comparison of other activation phenotypes showed significant differences between cord and adult samples; cord monocytes and mDCs demonstrated a higher frequency of CD40-CD86+ cells and cord pDCs showed a significantly higher frequency of CD40+CD86- cells (Table 1).

**Table 1:** Frequency of costimulatory molecules on innate cells after RV exposure.

Cell	Phenotype	Cord (n = 7)	Adult (n = 5)	p value
**Monocyte**				
** **	CD40+CD86+, mean (SD)	71.7(16)	90.8(10.4)	0.048
** **	CD40+CD86-, mean (SD)	0.8(0.4)	0.8(0.4)	NS
** **	CD40-CD86+, mean (SD)	25.4(15.7)	7.5(9.7)	0.049
** **	CD40-CD86-, mean (SD)	2.1(1.4)	0.9(0.9)	NS
**mDC**				
** **	CD40+CD86+, mean (SD)	82.2(8.5)	94.2(2.9)	0.009
** **	CD40+CD86-, mean (SD)	1.7(0.7)	2.1(1.2)	NS
** **	CD40-CD86+, mean (SD)	8.5(4.2)	2.3(1.2)	0.009
** **	CD40-CD86-, mean (SD)	7.6(4.8)	1.4(0.7)	0.02
**pDC**				
	CD40+CD86+, mean (SD)	72.7(4.4)	81.8(3.9)	0.004
	CD40+CD86-, mean (SD)	15.9(5.7)	7.7(1.5)	0.01
	CD40-CD86+, mean (SD)	5.7(3.1)	4.7(1.9)	NS
	CD40-CD86-, mean (SD)	5.7(3.7)	5.8(4.2)	NS

SD = standard deviation; NS = not significant.

### Expression of HLA-DR on RV exposed pDCs is comparable between cord and adults with greater increased fold-change in cord pDCs

**Fig 5:**
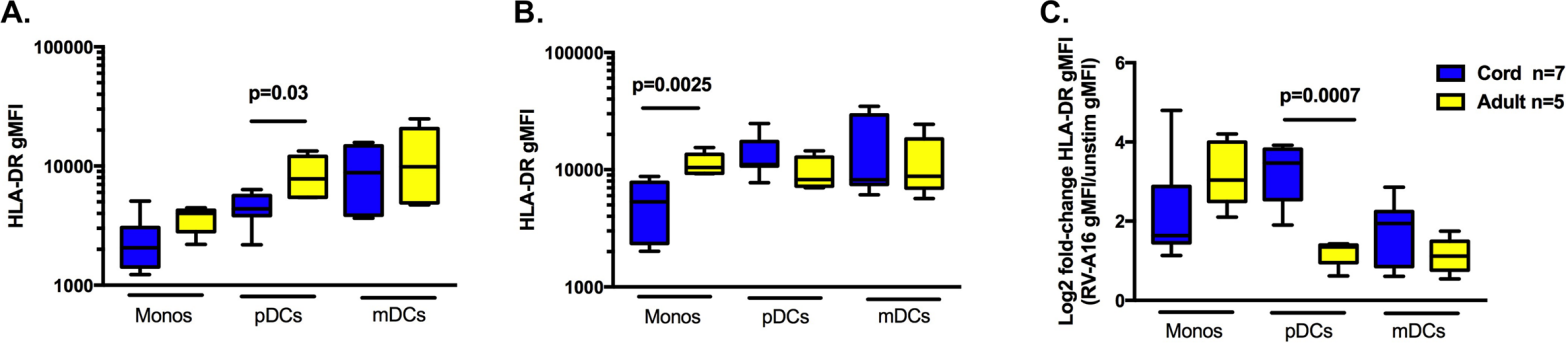
HLA-DR expression after RV stimulation. **A.** HLA-DR gMFI on monocytes, pDCs, and mDCs at rest (unstimulated). **B.** HLA-DR gMFI on monocytes, pDCs, and mDCs after 24 hours exposure to RV. **C.** Log2 of the ratio of RV HLA-DR gMFI/unstimulated HLA-DR gMFI. All box-and-whisker plots. Only signficant p values are shown for comparisons between adult and cord samples within a specific lineage (e.g. monocytes, pDCs, or mDCs).

To compare antigen presenting cell capacity of cord vs. adult cells, we measured HLA-DR gMFI. In unstimulated samples, the only significant difference in HLA-DR gMFI was in pDCs with adult cells greater than cord cells (Fig 5A). After 24 hours of RV stimulation, monocyte HLA-DR gMFI was significantly higher in adult cells compared to cord cells (Fig 5B). Measurement of the change between unstimulated and RV stimulation showed only pDC fold-changes were significantly different between adult and cord samples; cord pDCs demonstrated a greater change after RV stimulation compared to adult pDCs (Fig 5C).

### Cord B cells have increased expression of maturation markers compared to adult B cells after RV exposure

**Fig 6:**
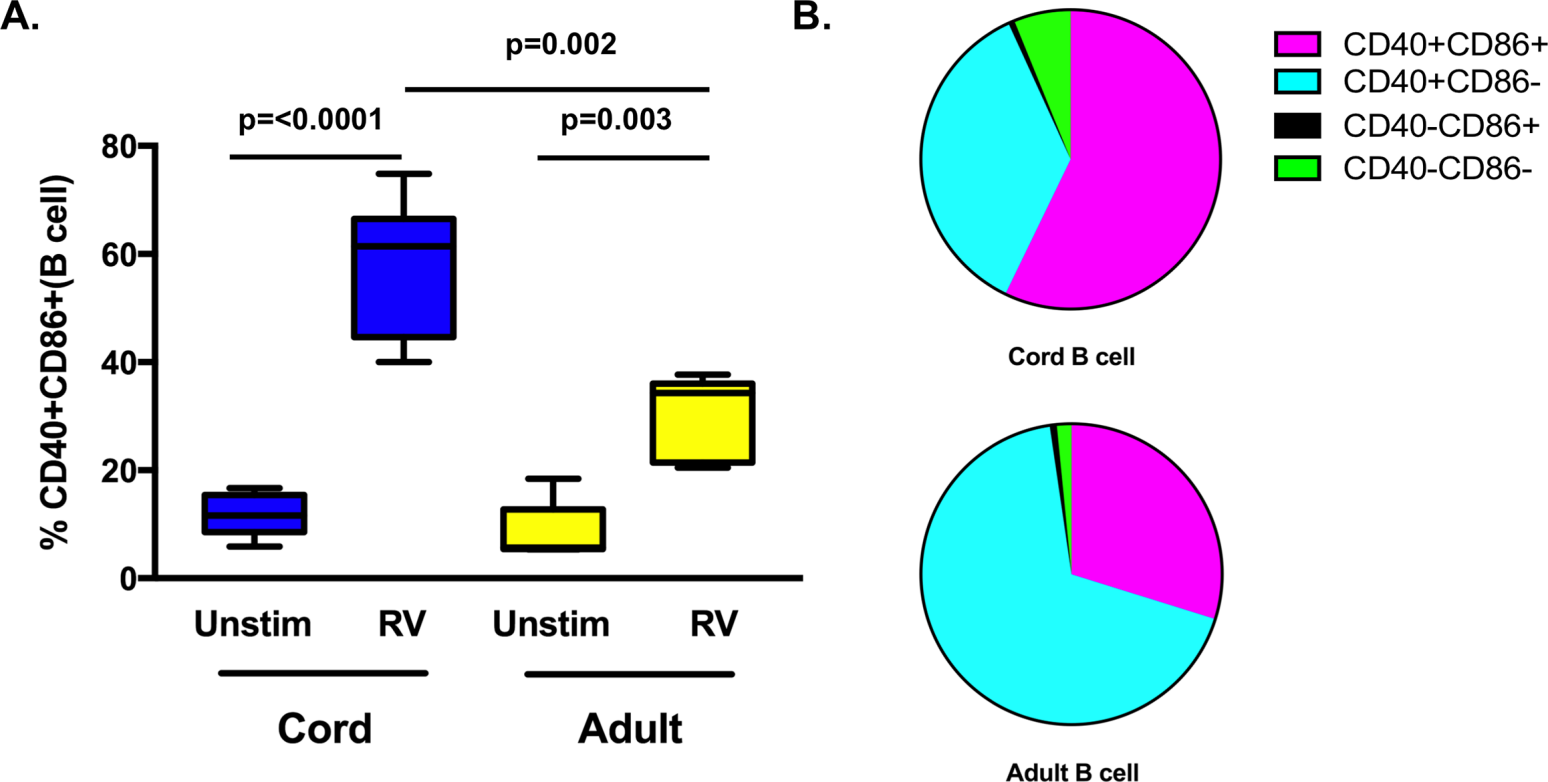
B cell responses after RV stimulation. **A.** Box-and-whisker plot of frequency of double positive (CD40+CD86+) B cells after 24 hours exposure to RV or unstimulated (Unstim). **B.** Parts of whole graph of varied phenotypes on right. Cord n = 7; Adult n = 5. Significant p values are shown in the graph, unstimulated comparisons between adult and cord samples were not significant.

In contrast to activation/maturation markers on monocyte, mDC, and pDC, RV exposure induced significantly increased CD40+CD86+ B cells on cord cells compared to adult B cells (Fig 6 and Table 2). After RV stimulation, most adult B cells had a CD40+CD86- phenotype (Table 2).

**Table 2:** Frequency of costimulatory molecules on B cells after RV exposure.

Phenotype	Cord (n = 7)	Adult (n = 5)	p value
CD40+CD86+, mean (SD)	57.2(13.2)	29.8(7.8)	0.002
CD40+CD86-, mean (SD)	36.1(11.6)	67.8(8.2)	0.0004
CD40-CD86+, mean (SD)	0.6(0.5)	0.8(0.9)	NS
CD40-CD86-, mean (SD)	6.1(2.3)	1.6(1.3)	0.003

SD = standard deviation; NS = not significant.

## Discussion

The immune underpinnings of increased respiratory viral illness severity during infancy remains poorly understood. Impaired anti-viral innate immune response has been primarily characterized by diminished IFN-alpha production and pDC frequency in neonates[1, 19, 24–28]. By defining activation and maturation phenotypes after RV exposure, our study extends these findings and includes a comparative analysis between adult and cord blood mononuclear cells. In addition to demonstrating diminished pDC polyfunctionality and IFN-alpha response in neonates, we found significant differences between cord and adult blood mononuclear cell activation/maturation markers. Finally, we found that RV induced different patterns of CD40 and CD86 on neonatal monocytes, mDCs and pDCs compared to B cells between cord and adult blood mononuclear cells.

Comparative studies of cord and adult blood dendritic cell frequency are conflicting [1, 29, 30]. One proposed immune explanation for increased severity of respiratory viral infections in infants is that the frequency of circulating pDCs is low [26–28]. Our study also found decreased mDC frequency in cord compared to adult samples. Since both mDCs and pDCs are important antigen-presenting cells for anti-viral T effector cell development, our findings suggest increased susceptibility to RV and other respiratory virus illness severity in neonates may be, in part, secondary to quantitative differences in pDCs and mDCs early in life [31, 32].

Another proposed immune risk factor for increased respiratory viral illness burden and severity susceptibility in both neonates and adults with asthma is impaired IFN-alpha production [1, 19, 24, 25, 31, 33, 34]. In addition to diminished IFN-alpha production, we show that RV-induced expression of TNF-alpha was also significantly diminished in cord blood pDCs. Neonatal pDCs had a higher frequency of TNF-alpha single positive cells compared to adult pDCs with the majority of adult pDCs producing both IFN-alpha and TNF-alpha. This finding emphasizes the value of flow cytometry in analyzing cell-specific cytokine responses. Studies are ongoing in our group to interrogate the relationship between polyfunctional pDCs and risk for severe respiratory viral infection.

RV can stimulate cytokine production from hematopoietic and bronchial epithelial cells via coordinated activation of endosomal TLR and non-endosomal pattern recognition receptors (PRRs) [35–37]. Thus, the observed diminished pro-inflammatory cytokine response to RV in neonatal pDCs suggests global diminished function including endosomal TLR and/or cytosolic PRRs (e.g. RIG-I and NOD). Two studies have reported that RV stimulation of PBMCs induces IL-6 secretion in an IFN-alpha independent manner [35, 38]. In our study, we only detected cytokine production in pDCs and IL-6 was not detected in adult or cord samples. This discrepancy is likely secondary to differences in incubation time as opposed to lack of assay sensitivity since these studies stimulated cells for ≥ 24 hours whereas our assay incubation time was 6 hours for intracellular cytokines. Previous studies focused on defining neonatal innate immune responses have typically relied on the use of surrogate stimulants of pathogens, such as TLR agonists. While this approach has provided important insights into the distinctions between the neonatal and adult immune system, these surrogates may not fully recapitulate effects of intact pathogens. There has been one previous comparative study using intracellular cytokine staining and a viral pathogen as an agonist in cord and adult blood samples. Marr et al. showed RSV infection of blood mononuclear cells demonstrated decreased type I interferon responses but intact pro-inflammatory cytokine production in cord blood samples compared to adult blood cells [1]. Gene expression analysis in adults infected in vivo with RV, RSV, or influenza revealed overlapping and non-overlapping gene signatures, a finding consistent with the differences between our findings with RV and those of Marr et al. with RSV [39]. A direct comparison between distinct respiratory viruses could better inform vaccine strategies and establish neonatal biomarkers for pathogen-specific disease risk.

Compared to the responses of adult cells, TLR stimulation of neonatal monocytes and mDCs resulted in decreased upregulation of costimulatory molecules [40–42]. In our study, RV upregulated costimulatory markers on a broad range of cells for both cord and adult samples. Unexpectedly, we found that RV induced increased expression of costimulatory markers (CD40 and CD86) on neonatal B cells compared to adult B cells. There are developmental distinctions between adult and cord blood B cells, but the distinctions with respect to pathogen responses have not been well characterized [30, 43–45]. It is possible that age-related differences in type I IFN influence the activation profile, or that these differences are due to intrinsic cellular pathways [46, 47]. For example, neonatal and adult B cells express similar levels of most TLRs, but TLR9 expression may be increased in cord blood cells [48, 49].

Strengths of our study include the cell-specific analysis of phenotypic and functional differences between cord and adult blood cells after exposure to an infectious pathogen. Limitations of the study include use of isolated blood mononuclear cells thus effects of granulocytes were not studied. In addition, our adult samples may have previous exposures to RV with memory T and B cell anti-RV responses that could directly or indirectly contribute to our observed differences when compared to cord blood samples, however, these responses are not likely to affect early innate anti-viral responses. Lastly, our study used one RV serotype, RV-A16, and thus our findings may not be generalizable to all RV serotype infections.

In conclusion, the high burden of respiratory viral infections during infancy emphasizes the need to better define the immune mechanisms of protection. The necessary immune protective factors against respiratory viral infections are still being debated. Both T and B cell effector functions have been associated with protection from infection burden with recent evidence highlighting the critical role of mucosal immunity and resident memory T cells [50–54]. Innate immune cell immaturity in neonates and its relationship to adaptive immune cell (e.g. T and B cell) effector function in response to RV infection for protection remain poorly defined. Identifying cell-specific differences in the immune response to RV in early life should enable future studies to test for correlations between newborn anti-viral responses to the risk of severe respiratory viral illnesses. In-depth studies of the cellular responses of neonates at risk for severe respiratory illnesses could lead to new therapeutic approaches to reducing the burden of respiratory illnesses in early childhood.

## Supporting information

S1 Table 24 hours RV-A16 stimulation flow panel. (DOCX)

S2 Table 6 hours RV-A16 stimulation flow panel. (DOCX)

## Abbreviations

PBMC: peripheral blood mononuclear cells
DC: dendritic cell
RV: rhinovirus
gMFI: geometric mean fluorescence intensity
RSV: respiratory syncytial virus
TLR: toll-like receptor
PRR: pathogen recognition receptor
IFN: interferon
IL: interleukin
NOD: nucleotide-binding oligomerization domain
RIG: retinoic acid-inducible gene
