# Supplementary material for: Neonatal immune response to rhinovirus A16 has diminished dendritic cell function and increased B cell activation
Source: PLoS One. 2017 Oct 18;12(10):e0180664. doi: 10.1371/journal.pone.0180664 (PMC5646756; doi:10.1371/journal.pone.0180664)
Supplement: S1 Table — (DOCX) [file pone.0180664.s001.docx]

**S1 Table: 24 hours RV-A16 Stimulation Flow Panel**

| **Specificity** | **Clone** | **Fluorophore** | **Notes** |
| --- | --- | --- | --- |
| CD40 | 5C3 | BV421 | Activation marker |
| CD123 | 9F5 | BV711 | pDC |
| CD86 | IT2.2 | BV510 | Activation marker |
| HLA DR | TU36 | FITC | Activation marker |
| FcεRIα | AER-37 | PE | Basophils, DC and monocytes |
| CD19 | HIB19 | PE-Cy5 | B cell |
| CD14 | M5E2 | PE-Cy7 | Monocytes |
| CD11c | S-HCL-3 | APC | mDC |
