# Supplementary material for: Neonatal immune response to rhinovirus A16 has diminished dendritic cell function and increased B cell activation
Source: PLoS One. 2017 Oct 18;12(10):e0180664. doi: 10.1371/journal.pone.0180664 (PMC5646756; doi:10.1371/journal.pone.0180664)
Supplement: S2 Table — (DOCX) [file pone.0180664.s002.docx]

**S2 Table: 6 hours RV-A16 Stimulation Flow Panel**

| **Specificity** | **Clone** | **Fluorophore** | **Notes** |
| --- | --- | --- | --- |
| CD14 | 61D3 | PE-Cy7 | Monocytes |
| CD11c | S-HCL-3 | APC | mDC |
| CD123 | 9F5 | PE | pDC |
| HLA-DR | TU36 | PE-Cy5 | Activation marker |
| IL-12/23 p40 | C8.6 | eF450 | Cytokine |
| TNF-α | Mab11 | A700 | Cytokine |
| IFN-α | A11 | FITC | Cytokine |
| IL-6 | MQ2-13A5 | APC-Cy7 | Cytokine |
